# Navigating the Challenges: A Comprehensive Review of Adolescent Gynecological Problems

**DOI:** 10.7759/cureus.56200

**Published:** 2024-03-14

**Authors:** Bhavana V Waghmare, Shubhada Jajoo

**Affiliations:** 1 Obstetrics and Gynaecology, Jawaharlal Nehru Medical College, Datta Meghe Institute of Higher Education & Research, Wardha, IND

**Keywords:** policy advocacy, healthcare access, reproductive education, psychosocial factors, menstrual disorders, adolescent gynecological health

## Abstract

This comprehensive review delves into the spectrum of adolescent gynecological problems, shedding light on the multifaceted challenges faced by individuals between the ages of 10 and 19. Covering normal developmental changes, common issues such as menstrual disorders, and the intricate interplay of psychosocial and cultural factors, the review provides a holistic understanding of adolescent gynecological health. Key findings underscore the importance of tailored education, destigmatizing reproductive health discussions, and recognizing the critical role of mental health in overall well-being. The conclusion issues a compelling call to action, urging healthcare providers to adopt patient-centered practices, educators to integrate comprehensive sexual education, and policymakers to advocate for inclusive policies. This review serves as a valuable resource, guiding collective efforts to enhance the well-being of adolescents as they navigate the challenges of gynecological health on their journey to adulthood.

## Introduction and background

Adolescent gynecological problems refer to health issues that specifically impact individuals between the ages of 10 and 19, during the pivotal stage of adolescence. These issues encompass a wide range of conditions affecting the female reproductive system, such as menstrual disorders, contraception, pelvic pain, and more. Understanding the unique nature of these problems is crucial for healthcare professionals, educators, and parents alike, as it enables the development of targeted interventions and support systems [1]. The significance of addressing adolescent gynecological health cannot be overstated. This period plays a pivotal role in laying the foundation for a woman's reproductive and overall well-being. Neglecting or dismissing the challenges that adolescents face in this realm can have lasting consequences on their health and quality of life. Comprehensive and sensitive care during this developmental stage not only addresses immediate concerns but also sets the stage for a lifetime of positive health outcomes [2].

Moreover, addressing adolescent gynecological health is intricately tied to promoting overall health literacy and empowerment. By providing young individuals with the knowledge and resources to understand and manage their reproductive health, we contribute to the development of informed and confident adults capable of making sound decisions about their well-being [2]. This review aims to encompass a broad spectrum of topics related to adolescent gynecological health. From exploring normal developmental changes during puberty to discussing common gynecological issues and reproductive anatomy, the scope of this review is comprehensive. Additionally, we will delve into psychosocial and cultural considerations, access to healthcare, preventive measures, and future directions in adolescent gynecological health. By delving into each facet of this multifaceted subject, we hope to provide a holistic understanding of the challenges faced by adolescents in the realm of gynecological health. Ultimately, this review aspires to serve as a valuable resource for healthcare professionals, educators, parents, and policymakers, fostering a collective effort to enhance the well-being of adolescents on their journey to adulthood.

## Review

Common adolescent gynecological issues

Menstrual Disorders

Menstrual irregularities: Among adolescent girls, menstrual irregularities are prevalent and can stem from a variety of factors. Research indicates that irregular menstrual cycles are more common in younger teens than older ones, with a higher proportion of younger teens reporting extended menstrual flow [3]. While irregular periods are often a normal aspect of teenage development, certain medications, excessive exercise, extreme fluctuations in body weight, or inadequate caloric intake can contribute to irregularities. Additionally, hormonal imbalances may also disrupt menstrual patterns [4]. Dysfunctional uterine bleeding (DUB) stands out as one of the primary concerns among adolescent girls, characterized by irregular and heavy menstruation [5]. Further menstrual disorders encompass dysmenorrhea, amenorrhea, polycystic ovary syndrome (PCOS), and hyperandrogenism [3,6,7]. Healthcare providers must discern abnormal menstrual patterns during adolescence to facilitate early detection of underlying conditions and administer appropriate treatment [6,7].

Dysmenorrhea: Dysmenorrhea, a term denoting painful menstrual periods, affects a significant portion of women, with over half experiencing discomfort lasting one to two days monthly [8]. This condition can be categorized as primary or secondary. Primary dysmenorrhea involves cramping pain preceding or coinciding with menstruation, attributed to natural chemicals known as prostaglandins produced in the uterine lining. Prostaglandins induce contractions in the uterus muscles and blood vessels, peaking during the initial day of menstruation and subsiding as the uterine lining sheds [8]. Secondary dysmenorrhea arises from conditions such as endometriosis, uterine fibroids, or pelvic inflammatory disease [9]. Managing dysmenorrhea symptoms typically entails prostaglandin inhibitors like nonsteroidal anti-inflammatory drugs (NSAIDs), oral contraceptives, progesterone, dietary modifications, and vitamin supplements [9]. Severe pain or interference with daily activities warrants consultation with a healthcare provider [8,9].

Amenorrhea: The absence of menstruation, termed amenorrhea, raises concerns among adolescent girls. Healthcare providers must differentiate between normal and abnormal menstruation patterns and possess insight into adolescent girls' menstrual cycles. Evaluation for primary amenorrhea is warranted for adolescents yet to experience menarche, with particular attention to assessing breast development by age 13 [6]. Various factors, including hormonal imbalances, eating disorders, excessive exercise, fluctuations in body weight, PCOS, and other medical conditions, can precipitate amenorrhea [10]. Identifying and addressing the underlying causes of amenorrhea in adolescents is crucial for their overall health and well-being [7].

Contraception and Sexual Health

Birth control methods: Access to contraception stands as a critical pillar in the realm of sexual and reproductive healthcare for adolescents. Healthcare providers must possess a comprehensive understanding of contraception options and their efficacy, regularly assess adolescents' sexual histories, and offer confidential and consent-based contraceptive care. Adolescents should receive counseling on and have access to a wide array of contraceptive services, with an emphasis on discussing the most effective methods first. Moreover, adolescents require access to accurate information and the ability to choose the contraception method that is safe, effective, affordable, and acceptable to them [11,12]. Several general methods of contraception exist, including barrier methods, hormonal methods, intrauterine devices (IUDs), and sterilization. Individuals must collaborate with their healthcare providers in selecting the most suitable method, considering factors such as overall health, age, frequency of sexual activity, and future childbearing desires [13]. While hormonal contraceptives and IUDs boast high efficacy in preventing pregnancy, it is crucial to note that they do not offer protection against sexually transmitted diseases (STDs), including HIV. Hence, consistent and correct use of male latex condoms is recommended to mitigate the risk of STD transmission [14]. When deliberating over the appropriate birth control method, individuals should weigh the advantages and disadvantages of each type. Notably, while certain hormonal birth control methods are highly effective in preventing pregnancy, they do not shield against STDs. Hence, incorporating condom use alongside other birth control methods is advised for comprehensive STD protection [15].

Sexually Transmitted Infections (STIs)

STIs encompass infections primarily transmitted through unprotected sexual contact, with some being transmissible during pregnancy, childbirth, and breastfeeding, or through infected blood or blood products. Left untreated, STIs can yield severe consequences, including neurological and cardiovascular complications, infertility, ectopic pregnancy, stillbirths, and heightened susceptibility to HIV. Symptoms of STIs commonly include vaginal or urethral discharge, genital ulcers, and lower abdominal discomfort. Given that many STIs are asymptomatic, individuals may harbor an infection unknowingly. Trichomoniasis, chlamydia, gonorrhea, and syphilis stand out among the most prevalent and treatable STIs. It is pivotal to recognize that STIs are highly contagious, with individuals capable of transmitting an infection unknowingly. Routine STI screenings and testing are recommended, particularly for sexually active individuals. Adoption of condoms or other STI prevention measures can significantly reduce the risk of infection [16]. Annually, an estimated 374 million new infections of curable STIs, chlamydia, gonorrhea, syphilis, and trichomoniasis, occur worldwide. Furthermore, more than 30 different bacteria, viruses, and parasites can be transmitted through sexual contact, encompassing vaginal, anal, and oral intercourse. Certain STIs can also be transmitted from the mother to the child during pregnancy, childbirth, or breastfeeding. Seeking medical attention upon experiencing STI symptoms or suspecting exposure to an STI is crucial [16].

Pelvic Pain

Causes and evaluation: Pelvic pain represents a prevalent issue with a myriad of potential causes, categorized into acute and chronic classifications. Acute pelvic pain typically persists for less than one month, while chronic pelvic pain persists for three to six months or longer [17,18]. Common culprits of acute pelvic pain include idiopathic pelvic pain, pelvic inflammatory disease, acute appendicitis, ovarian cysts, and endometriosis [18,19]. Chronic pelvic pain may stem from factors such as irritable bowel syndrome, musculoskeletal pelvic floor pain, and painful bladder [17]. The evaluation of pelvic pain necessitates a comprehensive history and physical examination. Patient history should encompass inquiries about triggering and relieving factors, the relationship between pain and menstrual cycles, urination, sexual activity, and bowel movements, as well as responses to prior treatments [17]. While imaging and laboratory findings may aid in diagnosis, they often yield inconclusive results [17]. Diagnostic laparoscopy may be warranted in certain cases to establish a diagnosis [18]. Crucially, excluding confusable conditions such as neoplastic disease, infection, trauma, and spinal pathology is essential [17]. A multidisciplinary approach can be beneficial in diagnosing and managing complex cases of pelvic pain, emphasizing early diagnosis and treatment to prevent complications and enhance the patient's quality of life [18].

Management Strategies

Management strategies for gynecological issues in adolescents vary depending on the specific condition. For adolescents with seizure disorders, anticonvulsant medication constitutes first-line treatment, with hormonal therapy serving as an adjunctive approach. Ongoing education for adolescents with seizure disorders is imperative, covering potential adverse pregnancy outcomes, contraceptive options, efficacy, and menstrual cycle education [20]. In cases of gynecologic pathologies in the pediatric population, surgical planning is typically overseen by a multidisciplinary team involving pediatric gynecologic oncologists and urologists. Preoperative counseling is obligatory and should engage parents and patients, if appropriate for their age. Treatment may involve adnexal, uterine, and peritoneal procedures [21]. For adolescents grappling with dysmenorrhea and endometriosis, therapeutic goals encompass symptom alleviation, disease progression suppression, and preservation of future fertility. Recommended treatment for endometriosis in adolescents entails conservative surgical therapy for both diagnosis and treatment, supplemented by ongoing suppressive medical therapy. NSAIDs constitute the cornerstone of pain management for adolescents with dysmenorrhea and endometriosis. Notably, adolescents with endometriosis should not undergo oophorectomy or hysterectomy [22].

PCOS

Definition and diagnosis: PCOS stands as a prevalent endocrine disorder impacting women of reproductive age. It manifests through a combination of signs and symptoms, including irregular menstrual cycles, excess androgen (male hormone) levels, and the presence of polycystic ovaries. The diagnosis of PCOS typically hinges on meeting at least two of the following criteria: irregular periods, indications of heightened androgen levels (such as excess body hair or acne), and the identification of polycystic ovaries through ultrasound imaging. It's imperative to rule out other conditions that could evoke similar symptoms, such as thyroid disorders. While the precise etiology of PCOS remains elusive, it is believed to involve a blend of genetic and environmental factors, encompassing insulin resistance and obesity [23,24]. Diagnosing PCOS necessitates a comprehensive approach, incorporating a detailed medical history, physical examination, and potentially additional assessments like ovarian ultrasound and hormone level measurements. Healthcare providers must adopt a holistic perspective in the diagnosis and management of PCOS, given the condition's multifaceted and nuanced nature [25]. Symptoms of PCOS can vary widely, encompassing irregular or absent menstrual cycles, excess body hair, acne, weight gain, and infertility. Early diagnosis and intervention, coupled with lifestyle adjustments like weight management, play pivotal roles in symptom management and mitigating the risk of long-term complications associated with PCOS [23].

Treatment approaches: Treatment strategies for PCOS encompass lifestyle modifications and tailored medications aimed at addressing individual symptoms. Lifestyle adjustments, including maintaining a healthy weight, regular exercise, and a balanced diet, form the cornerstone of PCOS management. Even modest weight reduction can yield significant improvements and enhance medication efficacy. Dietary strategies such as carbohydrate moderation and opting for complex carbohydrates, alongside physical activity, aid in managing blood sugar levels and promoting ovulation [23,26]. Medications assume a central role in symptom management for PCOS. Hormonal contraceptives are frequently prescribed to regulate menstrual cycles, diminish androgen levels, and alleviate acne. For individuals seeking conception, medications like clomifene may be recommended to induce ovulation, with metformin serving as an alternative if clomifene proves ineffective. Supplementary medications such as spironolactone and antiandrogens may be employed to tackle hirsutism and acne. Moreover, lifestyle modifications and weight loss are underscored due to their potential to enhance the endocrine profile and bolster ovulation and fertility prospects [27,28]. The management of PCOS entails a multifaceted approach, blending lifestyle adjustments with targeted pharmacotherapy to address diverse symptoms and optimize the overall health and well-being of individuals affected by PCOS.

Psychosocial and cultural considerations

Addressing Stigma and Taboos

Menstrual stigma remains a persistent issue affecting girls and women globally, with profound implications for personal, social, and economic well-being. Initiatives and policies to dismantle the silence surrounding menstruation and promote menstrual education should prioritize bodily autonomy and comprehensive menstrual health education [29]. The stigmatization of vulnerable adolescents, coupled with social taboos and fear of repercussions, often deters individuals from seeking assistance and may lead to concealing injuries. Adolescents, particularly in low- and middle-income countries, may encounter shame and stigma when accessing sexual and reproductive health (SRH) services [30,31].

Cultural norms and societal stigma surrounding STIs exacerbate concerns about confidentiality and community judgment, shaping provider behavior toward youth and contributing to feelings of stigma and shame. Addressing barriers to STI services may entail challenging cultural norms related to adolescent sexuality and enhancing clinic systems and provider attitudes [31]. Interventions targeting genital and menstrual stigma hold particular promise in enabling young women to access preventative care. Combating internalized body stigma is crucial for fostering positive body image and self-esteem among adolescents [32]. In addressing stigma and taboos within adolescent gynecological care, collaborative efforts among healthcare providers, policymakers, and educators are imperative. This entails raising awareness, advocating for body positivity, and delivering comprehensive education on menstrual health, sexual health, and reproductive health [29-32].

Cultural Influences on Adolescent Gynecological Health

Cultural beliefs and values play a significant role in shaping adolescents' attitudes toward SRH, as well as their parents' willingness to discuss SRH topics with them and the types of services they seek. For instance, in certain pastoral communities in Uganda, cultural practices surrounding sexuality can have profound implications for the SRH outcomes of adolescent girls [33]. Religious influences also exert considerable sway over adolescents' perspectives on premarital sex, the need for comprehensive sex education, and access to SRH services. In Ghana, religious norms promoting abstinence and condemning premarital sex can strongly influence the decision-making and behaviors related to adolescent SRH [34].

Gender norms, too, hold sway over adolescent SRH, with societal expectations regarding motherhood or perceptions of adolescence as a transitional life stage shaping approaches to SRH care and education. These norms can significantly impact adolescents' perceptions of their SRH needs and the services they feel comfortable accessing [34]. Healthcare providers must prioritize cultural competence, being attuned to the cultural factors that shape adolescents' SRH experiences. This awareness enables providers to understand their patients better and offer tailored services that address the unique needs of diverse populations [35]. Efforts to address cultural barriers to SRH service access among adolescents require multifaceted approaches. This may involve addressing clinic systems, cultivating provider attitudes that are sensitive to cultural nuances, and challenging cultural taboos surrounding adolescent sexuality. By doing so, the uptake of SRH services can be enhanced, and experiences of shame and stigma can be mitigated [31].

Mental Health Aspects

Adolescent gynecological health is intricately intertwined with mental well-being, necessitating healthcare providers' awareness of the mental health dimensions inherent in adolescent gynecological care. Mental health disorders are relatively prevalent among adolescents and can hinder their capacity to comprehend and articulate their gynecological health requirements [36,37]. Certain mental health conditions or their treatments may also disrupt the hypothalamic-pituitary-gonadal axis, thereby precipitating menstrual irregularities [36,37]. Adolescents grappling with mental illness may engage in risky sexual behavior, potentially leading to unintended pregnancies or STIs [36]. Managing pregnant adolescents with mental illness who require psychopharmacologic treatment poses a unique challenge, requiring careful consideration of the risks and benefits of medication usage [36].

Healthcare providers must be cognizant of the obstetric and gynecologic implications of mental health disorders and their treatments. They should be equipped to promptly refer patients, coordinate care, and address gynecologic side effects stemming from psychiatric medications [36,37]. Beyond mental health disorders, psychosocial and cultural factors also exert significant influence on adolescent gynecological health. These factors encompass societal beliefs, traditions, values, and practices that shape adolescents' perspectives on and approaches to SRH [38]. Cultural competence among healthcare providers is essential, as they must be attuned to the cultural dynamics shaping adolescents' SRH needs [38]. Healthcare providers must recognize the mental health dimensions inherent in adolescent gynecological care and understand the impact of psychosocial and cultural factors on adolescent SRH. They should be poised to offer timely referrals, coordinate care, and manage gynecologic side effects of psychiatric medications. Additionally, cultural competence is paramount, as providers must navigate and respect the cultural factors shaping adolescents' SRH needs [36-39].

Access to healthcare

Barriers to Healthcare for Adolescents

Confidentiality and privacy are paramount concerns for adolescents when seeking healthcare, particularly for sensitive issues like SRH [40,41]. Ensuring their medical information remains confidential and their privacy is respected can significantly impact their willingness to seek care and discuss their health concerns openly. Lack of awareness and knowledge poses another barrier for some adolescents who may not be fully aware of the available healthcare services or how to access them [40,42]. Providing comprehensive information about healthcare resources and pathways to access care ensures adolescents can navigate the healthcare system effectively.

Stigma and embarrassment often deter adolescents from seeking healthcare, especially for services related to SRH [40]. Overcoming feelings of stigma and embarrassment requires creating supportive, non-judgmental, and sensitive environments to adolescents' unique needs and concerns. Financial barriers can also impede adolescents' access to healthcare services, particularly for those with limited financial resources [43]. Addressing financial barriers may involve providing affordable or free healthcare options and ensuring that adolescents are aware of available financial assistance programs.

**Figure 1:**
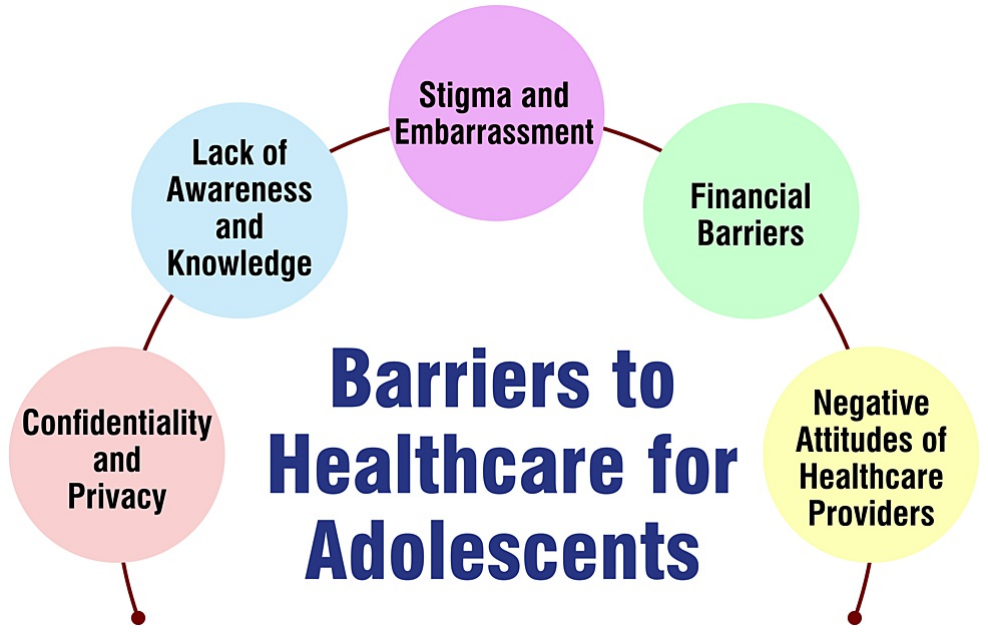
Barriers to healthcare for adolescents

Negative attitudes of healthcare providers can further compound barriers to adolescent care, impacting their willingness to seek help [41]. Healthcare providers must adopt respectful, supportive, and youth-friendly attitudes to encourage adolescents to engage with healthcare services. Addressing these barriers requires a multifaceted approach that prioritizes confidentiality, provides comprehensive information, reduces stigma, addresses financial barriers, and fosters positive attitudes among healthcare providers [43]. By implementing such strategies, healthcare systems can improve the accessibility and effectiveness of healthcare services for adolescents. Barriers to healthcare for adolescents are shown in Figure 1.

Importance of confidentiality

Confidential care for adolescents is pivotal in encouraging access to healthcare services and fostering discussions about sensitive topics and behaviors [44]. Adolescents who trust that their information will be kept confidential are more likely to seek medical assistance, knowing their privacy will be respected. Moreover, confidentiality serves as a cornerstone principle in healthcare interactions between providers and adolescent patients, promoting open communication and facilitating honest discussions about health concerns, particularly sensitive issues related to SRH [45]. This open dialogue is essential for addressing adolescent health needs effectively.

Confidentiality also safeguards adolescents' personal and private information disclosed to healthcare providers [44]. This trust is fundamental for establishing a robust therapeutic relationship and delivering quality care tailored to each patient's individual needs. Additionally, confidentiality in adolescent healthcare acknowledges and addresses the concerns of mature minors, with some clinicians advocating for the assessment and documentation of their maturity in health records [45]. This approach enables healthcare providers to meet the unique needs of mature minors while safeguarding their privacy.

**Figure 2:**
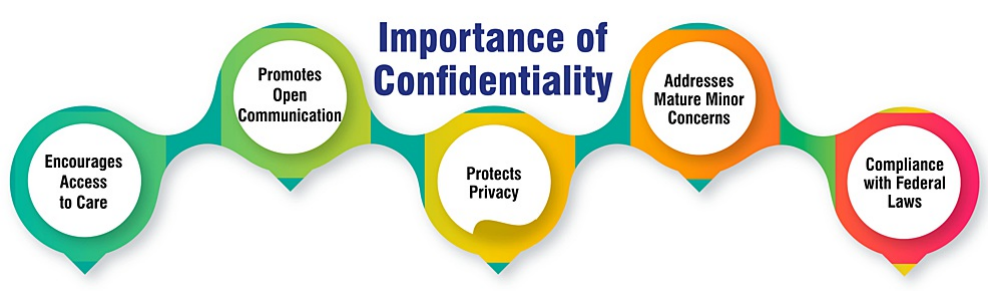
Importance of confidentiality

Furthermore, confidentiality aligns with federal laws aimed at protecting the privacy of minors [46]. Healthcare providers must adhere to these laws and uphold confidentiality standards to ensure compliance and protect the rights of adolescent patients. Confidentiality is critical to adolescent healthcare, facilitating access to care, promoting open communication, safeguarding privacy, addressing mature minor concerns, and complying with federal laws [44-47]. Prioritizing confidentiality ensures that adolescents receive the comprehensive and respectful care they deserve. The importance of confidentiality is shown in Figure 2.

Role of School and Community Health Programs

School and community health programs are instrumental in advancing the health and well-being of adolescents. One such initiative is school-based healthcare (SBHC) programs, which offer accessible sexual, reproductive, and mental healthcare services tailored to adolescents' needs [48]. SBHCs not only deliver care directly to schools but also mitigate obstacles by minimizing transport costs and enhancing accessibility [49]. Furthermore, community health centers can function as SBHCs, delivering comprehensive primary care services to underserved children and youth [50].

In addition to healthcare provision, school health services offer systems for identifying and addressing students' health and educational challenges and delivering comprehensive and pertinent health education [50]. These programs are pivotal in diminishing the prevalence of health risk behaviors among youth and fostering improved academic performance [51]. Moreover, they contribute to fostering healthy, safe, and supportive school environments, implementing effective policies and initiatives to prevent HIV, STDs, and unintended pregnancies while also expanding youth access to sexual health services [51]. Overall, school and community health programs are indispensable in championing the health and well-being of adolescents. These initiatives, through the provision of accessible healthcare services, reducing barriers to care, and establishing nurturing environments, are essential components of adolescent health promotion efforts.

Preventive measures and health promotion

Importance of Regular Check-Ups

During check-ups, healthcare providers monitor adolescents' growth, development, and overall health, employing measurements such as weight, height, and body mass index (BMI) to ensure they progress as expected [52]. These routine assessments enable the early identification of deviations from the norm, allowing for timely intervention if necessary. Moreover, check-ups serve as a platform for healthcare providers to conduct age-appropriate screenings and examinations, facilitating the detection of potential health issues [52,53]. This comprehensive approach encompasses screening for conditions like high blood pressure, diabetes, and high cholesterol and indicators of mental health disorders such as depression, enhancing the likelihood of early intervention and management.

Adolescents can also receive vaccinations and immunizations during check-ups, protecting them against serious illnesses [54,55]. By staying up-to-date with their immunizations, adolescents maintain community health and safeguard their well-being. Additionally, check-ups allow healthcare providers to deliver health education, address concerns, and offer counseling on various topics, including nutrition, exercise, sexual health, substance abuse, and mental health [52,53,55]. This educational component empowers adolescents to make informed decisions about their health and adopt healthy behaviors. Furthermore, check-ups play a crucial role in promoting self-care skills among adolescents, encouraging them to take ownership of their health and well-being [53]. By instilling habits of self-care and responsibility during adolescence, healthcare providers lay the foundation for lifelong health management and disease prevention.

Education on Healthy Lifestyles

Encouraging healthy lifestyles among adolescents is paramount for their overall well-being. Adopting regular exercise, a balanced diet, sufficient rest, and effective stress management is crucial for disease prevention and fostering positive health outcomes [56]. Evidence suggests educational programs promoting healthy lifestyles can ameliorate symptoms and mitigate adolescent cardiovascular risk factors [57]. These initiatives typically underscore the significance of physical activity, nutritional balance, injury prevention, and stress reduction [58]. Numerous endeavors, including initiatives like the Healthy Lifestyles program, furnish educational materials and engagement opportunities to empower students and families in making informed health decisions [59]. Embracing a comprehensive approach to health, these programs equip individuals with the tools and skills to bolster confidence and embark on a journey toward a healthy, self-reliant, and fulfilling lifestyle [60]. Education on healthy lifestyles is indispensable for adolescents and should prioritize instilling behaviors such as regular exercise, balanced nutrition, injury prevention, and stress management. Such endeavors wield significant influence over young individuals' long-term health and well-being.

Immunizations and Screenings

Immunizations and screenings constitute pivotal components of preventive healthcare for adolescents. Immunizations, commonly called vaccinations, play a critical role in safeguarding adolescents against infectious diseases. The US Centers for Disease Control and Prevention (CDC) advocates routine vaccination for children, adolescents, and adults as a preventive measure against vaccine-preventable diseases [61]. Conversely, screening entails medical tests conducted to detect specific disorders before symptoms manifest, facilitating early detection when treatment may be more effective [61]. Before administering vaccines, thorough screening of patients for contraindications and precautions is imperative to prevent adverse reactions [62]. Screening tests and immunizations are recommended for adolescents to identify health conditions early and shield them from a spectrum of infectious diseases [63,64]. Immunizations and screenings are indispensable for the health and well-being of adolescents, acting as pivotal measures to prevent infectious diseases and facilitate the early detection of potential health concerns [63,64].

Future directions in adolescent gynecological health

Research Needs and Gaps

Research gaps in adolescent gynecological health have been identified across various domains, indicating areas for further investigation and attention. One such gap lies in the scarcity of data and research concerning the sexual and reproductive behaviors of adolescents in developing regions, as well as the associated health and economic ramifications. Additionally, there exists a notable disparity in the availability of SRH information, particularly for marginalized groups such as unmarried or never-married women in certain regions. Furthermore, there is a pressing need for more research delving into the societal factors that drive adolescents to desire pregnancy, alongside exploring the underlying reasons for the unmet need for contraception among young individuals [65]. Moreover, it has been underscored that children and adolescents possess unique gynecological needs, often overlooked by pediatricians and adult OB/GYNs, indicating a significant gap in care that necessitates attention [66]. These identified research gaps underscore the imperative for further investigation and focus on adolescent gynecological health. By addressing these gaps, healthcare providers and researchers can ensure the delivery of comprehensive and tailored care specifically designed to meet the needs of this population.

Innovations in Healthcare Delivery

Next-generation sequencing (NGS) technology has revolutionized the diagnosis of genetic diseases, offering faster and more accurate insights into patients' conditions. This advancement enables healthcare professionals to tailor personalized treatment plans, ultimately enhancing patient outcomes [67]. Similarly, 3D printing technology has opened new avenues in healthcare by allowing the creation of customized medical devices such as prosthetics, dental appliances, and surgical tools. These devices are precisely tailored to meet the unique needs of individual patients, improving treatment efficacy and patient satisfaction [67]. Immunotherapy represents a groundbreaking approach to treating cancer and autoimmune diseases. By harnessing the patient's immune system to combat disease-causing cells, immunotherapy offers promising prospects for improved treatment outcomes and prolonged survival rates [67]. Artificial intelligence (AI) is increasingly integrated into various healthcare processes, from diagnostics to treatment planning and patient monitoring. AI-driven solutions enhance accuracy and efficiency, improving patient care [67].

Point-of-care diagnostics transform healthcare by enabling immediate testing and diagnosis of medical conditions. This capability facilitates prompt treatment decisions, improving patient outcomes [67]. Virtual healthcare, including telemedicine and remote monitoring technologies, reshapes patient care delivery. These solutions enhance patient access and convenience while reducing the need for in-person visits [67]. Value-based care (VBC) models incentivize healthcare providers to prioritize high-quality care and patient outcomes over the volume of services provided. This shift in focus fosters improved patient care experiences and reduces overall healthcare costs [67]. Healthcare organizations are establishing innovation centers dedicated to creating new services, enhancing patient care, and reducing costs. These centers are hubs for fostering innovation and advancing healthcare delivery [68]. Technology giants like Amazon, Apple, Google, IBM, and Microsoft are investing in healthcare innovations to disrupt traditional delivery models. Their efforts focus on data-driven care delivery and consumer-centric solutions to improve patient experiences and outcomes [69]. Moreover, clinical trial data utilization advancements are informing more personalized and effective treatment approaches. These new data sources and tools aid clinical trial design, treatment decision-making, and ongoing patient care, ultimately enhancing treatment efficacy and patient outcomes [67]. By embracing and integrating these innovations into healthcare delivery models, healthcare providers can drive improvements in patient outcomes, cost reduction, and overall healthcare experiences.

Advocacy for Improved Policies

Promoting comprehensive sex education is crucial for ensuring that all youth have access to accurate information about SRH. Establishing national standards for comprehensive sex education in schools can help achieve this goal by providing medically accurate education on various topics, including anatomy, sexual development, gender identity, sexual behavior, STI prevention, and reproductive healthcare [70]. Supporting adolescent access to evidence-based, medically accurate SRH services is essential for ensuring that young people receive timely and comprehensive care. This includes incentivizing the provision of clinical services such as STI screening and treatment, contraception, pregnancy testing and care, pregnancy options counseling, and abortion services in outpatient clinics [70].

Encouraging the FDA to review oral contraceptives for over-the-counter use can help improve access to contraception for individuals of all ages. By allowing oral contraceptives to be available over the counter, individuals would have easier access to emergency contraception, regardless of age or insurance coverage, and without cost-sharing [70]. Reducing access disparities is paramount in ensuring equitable access to SRH services. Advocating for policies that expand available services and providers for underserved populations and reduce discriminatory barriers can help address disparities in access to care for marginalized communities [71]. Opposing political interference in healthcare is essential for safeguarding physicians' ability to provide evidence-based, compassionate care to their patients. Advocating against legislation that undermines physicians' autonomy, regulates or criminalizes abortion care providers, and imposes unjustified restrictions on access to care is crucial for protecting patients' rights and ensuring access to factual, individualized care and counseling [71].

## Conclusions

Our exploration of adolescent gynecological problems has illuminated the intricate facets of this critical aspect of young individuals' health. From the nuanced changes during puberty to the complexities of menstrual disorders, psychosocial considerations, and cultural influences, it is evident that a holistic approach is essential. Recapitulating our key findings underscores the pressing need for tailored education, the destigmatization of reproductive health discussions, and an acknowledgment of the pivotal role played by mental health in overall well-being. As we wrap up this review, a resounding call to action emerges. Healthcare providers must adopt patient-centered practices, educators should integrate comprehensive sexual education into curricula, and policymakers need to advocate for inclusive policies that prioritize adolescent gynecological health. Together, we can dismantle barriers, foster understanding, and pave the way for a future where adolescents navigate the challenges of gynecological health with informed confidence and resilient well-being.
